# Sotetsuflavone suppresses invasion and metastasis in non-small-cell lung cancer A549 cells by reversing EMT via the TNF-α/NF-κB and PI3K/AKT signaling pathway

**DOI:** 10.1038/s41420-018-0026-9

**Published:** 2018-02-14

**Authors:** Shaohui Wang, Yu Yan, Zhekang Cheng, Yanlan Hu, Tongxiang Liu

**Affiliations:** Key Lab of Ministry of Education, National Research Center on Minority Medicine, Minzu University of China, Beijing, 100081 People’s Republic of China

## Abstract

Epithelial-mesenchymal transition (EMT) is associated with tumor invasion and metastasis, and offers insight into novel strategies for cancer treatment. Sotetsuflavone was isolated from *Cycas revolute*, which has excellent anticancer activity in the early stages. The present study aims to evaluate the anti-metastatic potential of sotetsuflavone in vitro. Our data demonstrated that sotetsuflavone inhibits metastasis of A549 cells, and EMT. This inhibition was reflected in the upregulation of E-cadherin, and downregulation of N-cadherin, vimentin, and Snail. Mechanistically, our study demonstrated that HIF-1α played an important role in the anti-metastatic effect of sotetsuflavone in non-small-cell lung cancer A549 cells. Sotetsuflavone not only mediated VEGF expression but also downregulated VEGF and upregulated angiostatin, and simultaneously affected the expression of MMPs and decreased MMP-9 and MMP-13 expression. More importantly, HIF-1α expression may be regulated by the inhibition of PI3K/AKT and TNF-α/NF-κB pathways. These results suggest that sotetsuflavone can reverse EMT, thereby inhibiting the migration and invasion of A549 cells. This process may be associated with both PI3K/AKT and TNF-α/NF-κB pathways, and sotetsuflavone may be efficacious in the treatment of non-small-cell lung cancer.

## Introduction

Lung cancer is a malignant tumor with the highest morbidity and mortality worldwide, with 1.38 million new cases each year, and over one million deaths worldwide annually^1^. Non-small-cell lung cancer (NSCLC) accounts for 80–85% of lung cancers, with a 5-year survival rate of <15%^2,3^. Although advancements have been made in chemotherapy and radiotherapy, the overall survival rate of patients has remained unchanged in recent years^4^. In NSCLC patients, about 30% of patients have distant metastasis upon diagnosis, 50–60% of patients have distant metastasis during treatment, and eventually lung cancer metastasis can claim the lives of 80–90% of patients^5^. Therefore, inhibition and prevention of metastasis is key to the treatment of NSCLC^6^. *Cycas revolute* possess the function of reducing fever, preventing bleeding, and dissipating congestion. The chemical ingredients of *C. revolute* are mainly flavonoids^7,8^. Sotetsuflavone was isolated from *C. revolute* in our laboratory. Early experimental studies revealed that sotetsuflavone can inhibit A549 cell growth, but its mechanism remains to be researched.

Epithelial-mesenchymal transition (EMT) is a critical process of tumor invasion and metastasis^9^. Such changes enhance tumor cell migration, metastasis, and establishment of distant secondary tumors^10,11^. EMT is a dynamic and complex process inseparable from the interaction between multiple growth factors, protein molecules, transcription factors, and the pathways they regulate^12^. Hypoxia-inducible factor-1α (HIF-1α) plays an important role in tumor cell invasion, metastasis, immortalization, tumor angiogenesis, and cancer function^13^. Nuclear factor-κB (NF-κB) usually exists in the form of a p65-p50 dimer, and can induce transcription of related genes involved in immune responses, inflammatory responses, cell growth, development, division, and apoptosis^14^. Angiogenesis and the adaptation of cells to hypoxia are key to the development of tumors. Hypoxia can promote angiogenesis factors, including increasing vascular endothelial growth factor (VEGF), while also lowering angiogenesis inhibitory factors such as angiostatin^15^. Matrix metalloproteinases (MMPs) are important proteolytic enzymes involved in the degradation of the extracellular matrix (ECM) and basement membrane^16^. Their structure, function, and regulatory levels are closely linked to tumor invasion and metastasis^16^. Tumor necrosis factor-α (TNF-α) is an important cytokine that participates in inflammation, and the dynamic balance of immune cells and tumor progression^17^. NF-κB and STAT3 gradually become the core molecules involved in inflammatory-induced metastasis in a large number of molecular signaling pathways associated with inflammation and EMT^12^. Snail is a transcription factor that can stimulate the occurrence of EMT, which is significantly upregulated in various tumors and inhibits the expression of E-cadherin^18^. The reduction and deletion of E-cadherin expression can lead to the disappearance of cell polarity and decrease cell adhesion, and is an important marker of the processes involved in EMT^19^. The expression of E-cadherin is negatively correlated with tumor invasiveness, and the disappearance of epithelial cell polarity is accompanied by an increase in the expression of mesenchymal cell markers vimentin, N-cadherin, and fibronectin. Accordingly, this contributes to the occurrence of EMT processes, leading to the initiation of metastasis^20,21^. After tumor cell hypoxia, the expression of invasion-related proteins, such as snail, slug, fibronetin, and vimentin increased, while E-cadherin decreased^22^. Moreover, the invasion ability of tumor cells was enhanced. The tumor was prone to distant metastasis and had a deeper depth of tumor invasion^22^. The active phosphorylated NF-κB in the nucleus can bind to the promoter region of the *Snail-1α* gene via chromatin, which becomes the upstream activator of Snail-1α^23^. TNF-α can be activated by the NF-κB pathway and induce snail expression^23^. An imbalance in the PI3K/Akt pathway plays a key role in the formation and development of lung cancer, and the activation of the PI3K/Akt pathway can activate the transduction of several downstream signals and promote the development of NSCLC^24^.

Therefore, we evaluated the effects of sotetsuflavone on the migration and invasion of human NSCLC cell line A549. We further explored the possible mechanism of sotetsuflavone against NSCLC and provided a new theoretical basis for the treatment of lung cancer with Chinese medicine.

## Results

### Sotetsuflavone inhibited the migration and invasion of A549 cells

**Fig. 1:**
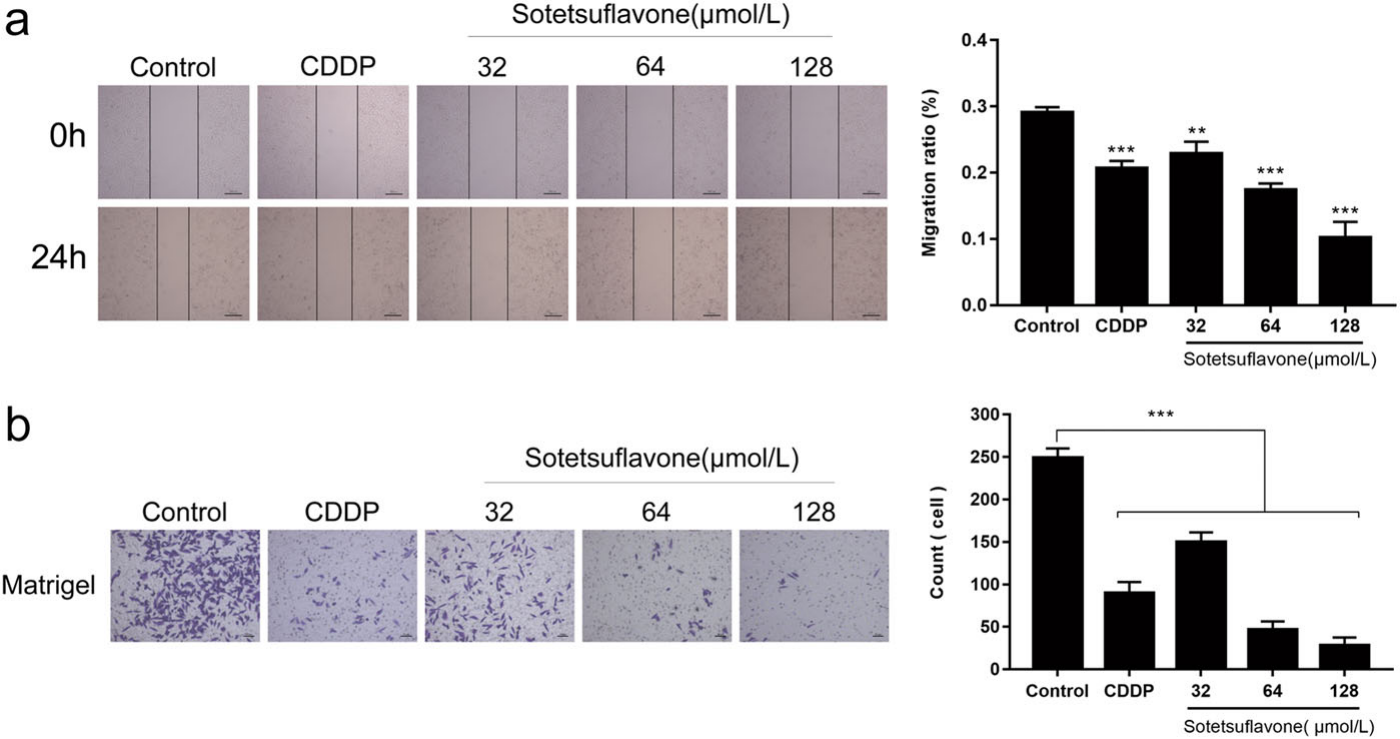
Sotetsuflavone inhibited migration and invasion of A549 cells. Based on the preliminary study, A549 cells were treated with 0, 32, 64, 128 μmol/L, and 10 μg/ml of CDDP for 24 h. **a** Wound-healing assay measured the migration ability changes. Original magnification ×40. **b** Transwell-matrigel invasion assay measured changes in invasive ability. Original magnification ×100. The results from three independent experiments were expressed as mean ± SD compared with the control group, **P < 0.01, ***P < 0.001. Combined with pre-experimental basis and the results of migration and invasion experiments, we finally selected 24 h as the follow-up experimental treatment time. The subsequent experimental concentration adjusted to was 0, 64, and 128 μmol/L

The scratch test and Transwell invasion assay demonstrated that sotetsuflavone inhibited cell migration compared to the control group. The sclerosis width gradually increased, and sclerosis on both sides of the cell density gradually reduced. At the same time, the scratch-healing rate of the sotetsuflavone treatment group decreased gradually in a dose-dependent manner (Fig. 1a). Interestingly, the invasion ability of A549 cells treated with different concentrations of sotetsuflavone was also significantly decreased, which demonstrated a significant dose-dependent inhibition of A549 cell invasion (Fig. 1b).

### Sotetsuflavone inhibited EMT in A549 cells

**Fig. 2:**
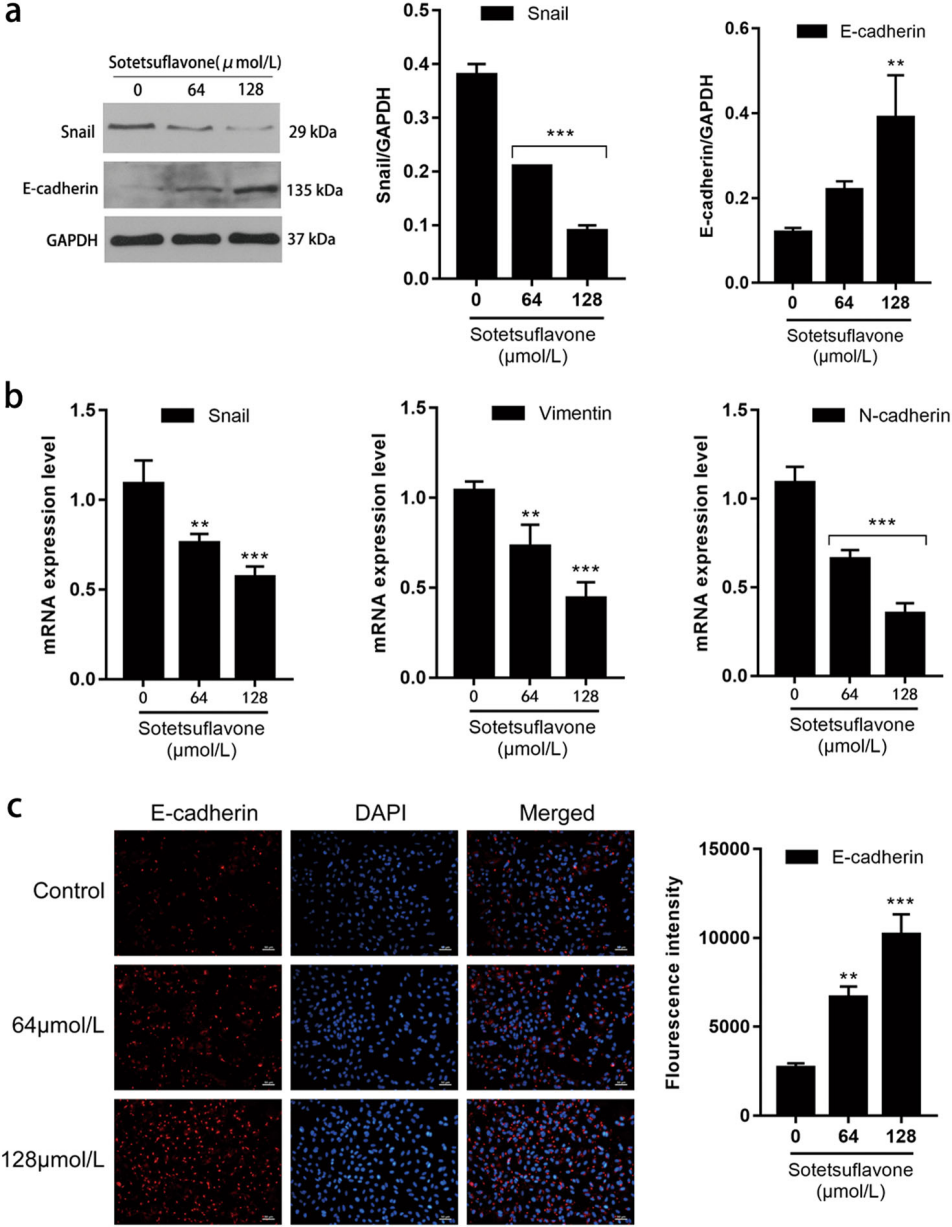
Sotetsuflavone inhibited EMT in A549 cells. **a** The expression of E-cadherin and snail protein as shown by western blotting. One gel (gel A) was run and blotted, and the blot was cut horizontally. The upper part was probed with anti-E-cadherin and anti-snail, and the lower part was probed with anti-GAPDH. **b** The expression of *snail*, *vimentin*, and *N-cadherin* identified by RT-PCR. **c** Immunofluorescence assay detected the expression of E-cadherin. Scale bar 50 μm. The data in the histogram represented the mean ± SD from three independent experiments (**P < 0.01, ***P < 0.001)

Sotetsuflavone significantly upregulated the expression of E-cadherin, and downregulated the expression of N-cadherin, vimentin, and snail, thereby inducing mesenchymal-epithelial transition (MET) (Fig. 2a–c). The results demonstrated that sotetsuflavone could inhibit the migration and invasion of A549 cells by reversing EMT.

### Sotetsuflavone inhibited HIF-1α, VEGF, angiostatin, MMP-9, and MMP-13 expression

**Fig. 3:**
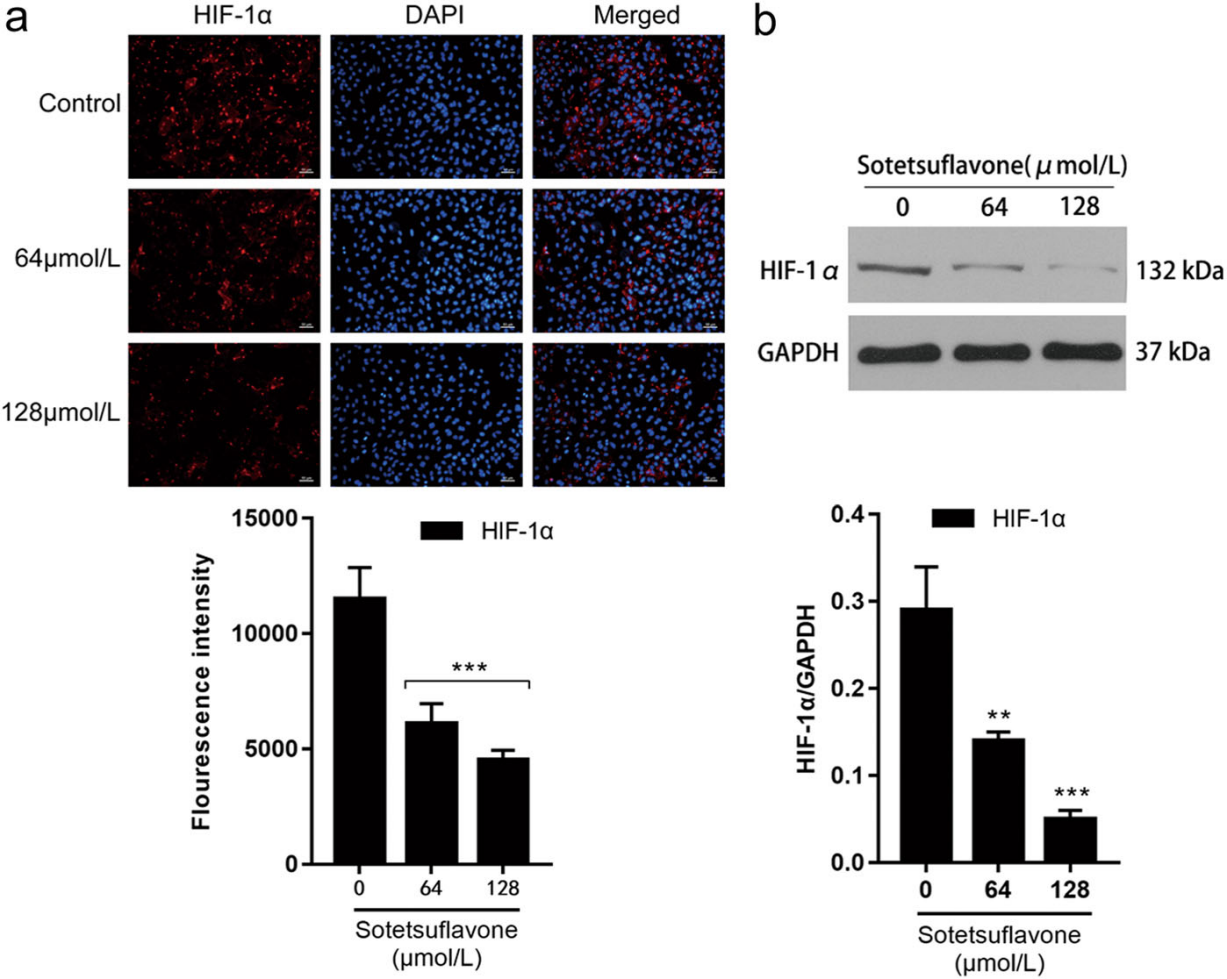
Sotetsuflavone inhibited HIF-1α expression. **a** Immunofluorescence assay detected the expression of HIF-1α. Scale bar 50 μm. **b** Western blotting detected HIF-1α expression. Using the same sample lysates as used for Fig. 2a, another gel (gel B) was run and probed with anti- HIF-1α. The GAPDH loading control is from gel A, and is the same as shown in Fig. 2a. The data in the histogram represent the mean ± SD from three independent experiments (**P < 0.01, ***P < 0.001)

HIF-1α mediates cell response to hypoxia, and HIF-1α protein overexpression has been reported in several solid tumors and their metastases^25^. We detected HIF-1α expression by western blot and immunofluorescence assay and discovered that sotetsuflavone decreased HIF-1α expression (Fig. 3a, b).

**Fig. 4:**
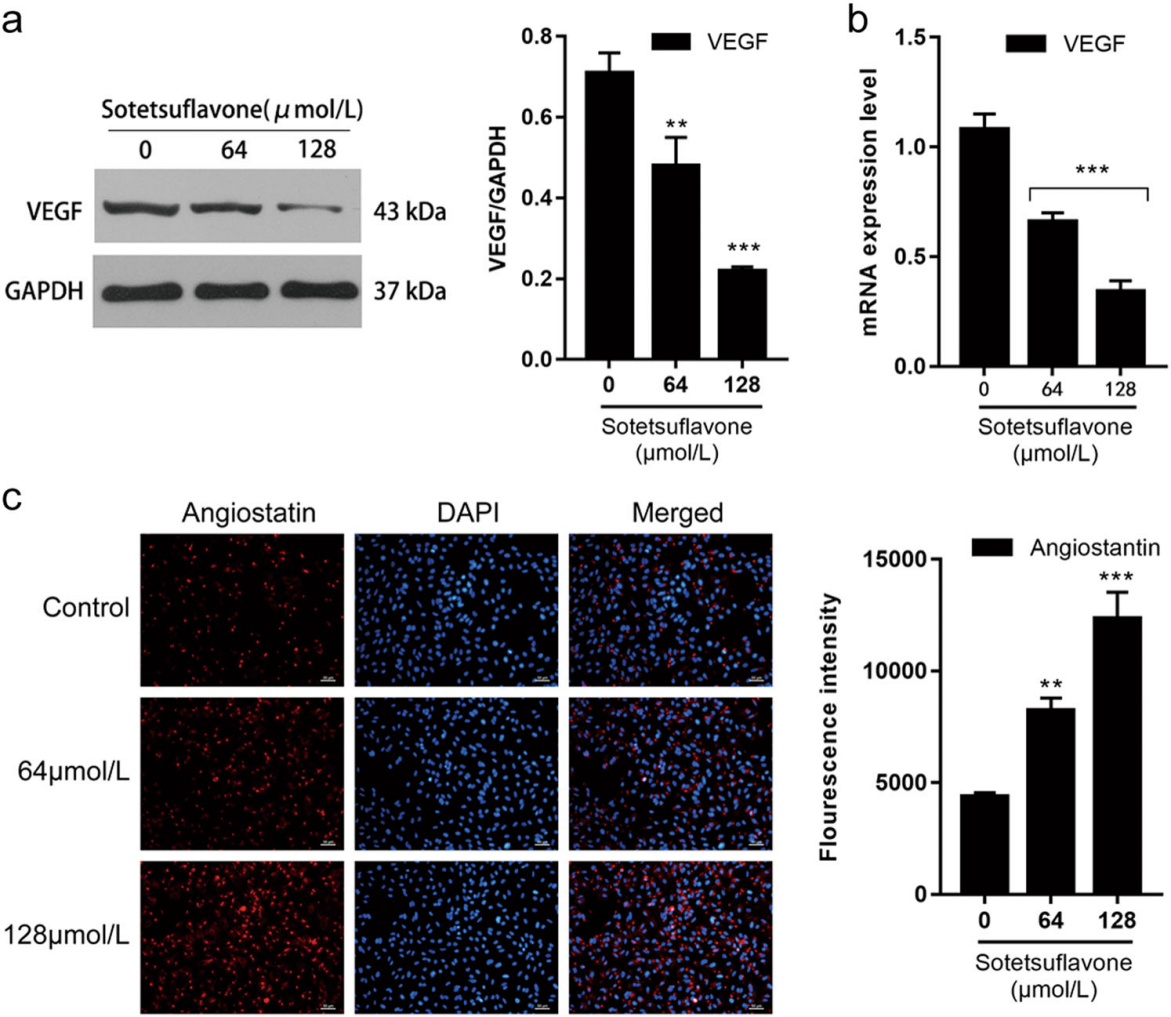
Sotetsuflavone inhibited VEGF and angiostatin expression. **a** Western blotting detection of VEGF protein expression. Using the same sample lysates as used for Fig. 2a, another gel (gel C) was run and probed with anti-VEGF. The GAPDH loading control is from gel A, and is the same as shown in Fig. 2a. **b** The expression of *VEGF* gene detected by RT-PCR. **c** Immunofluorescence assay detected the expression of angiostatin. Scale bar 50 μm. The data in the histogram represented the mean ± SD from three independent experiments (**P < 0.01, ***P < 0.001)

HIF-1α can mediate VEGF expression, and VEGF is an important factor in promoting angiogenesis and participating in tumor angiogenesis^26^. Western blotting analysis demonstrated that sotetsuflavone inhibited VEGF expression. Reverse transcription-PCR (RT-PCR) analysis further demonstrated that VEGF was downregulated in A549 cells treated with different concentrations of sotetsuflavone for 24 h. Angiostatin is an important angiogenesis inhibitory factor; we used the immunofluorescence assay to analyze the expression of angiostatin. Our results demonstrated that sotetsuflavone increased angiostatin expression. In combination with the results shown in Fig. 3, we concluded that sotetsuflavone could reduce VEGF expression and downregulate angiostatin expression by decreasing HIF-1α expression (Fig. 4a–c).

**Fig. 5:**
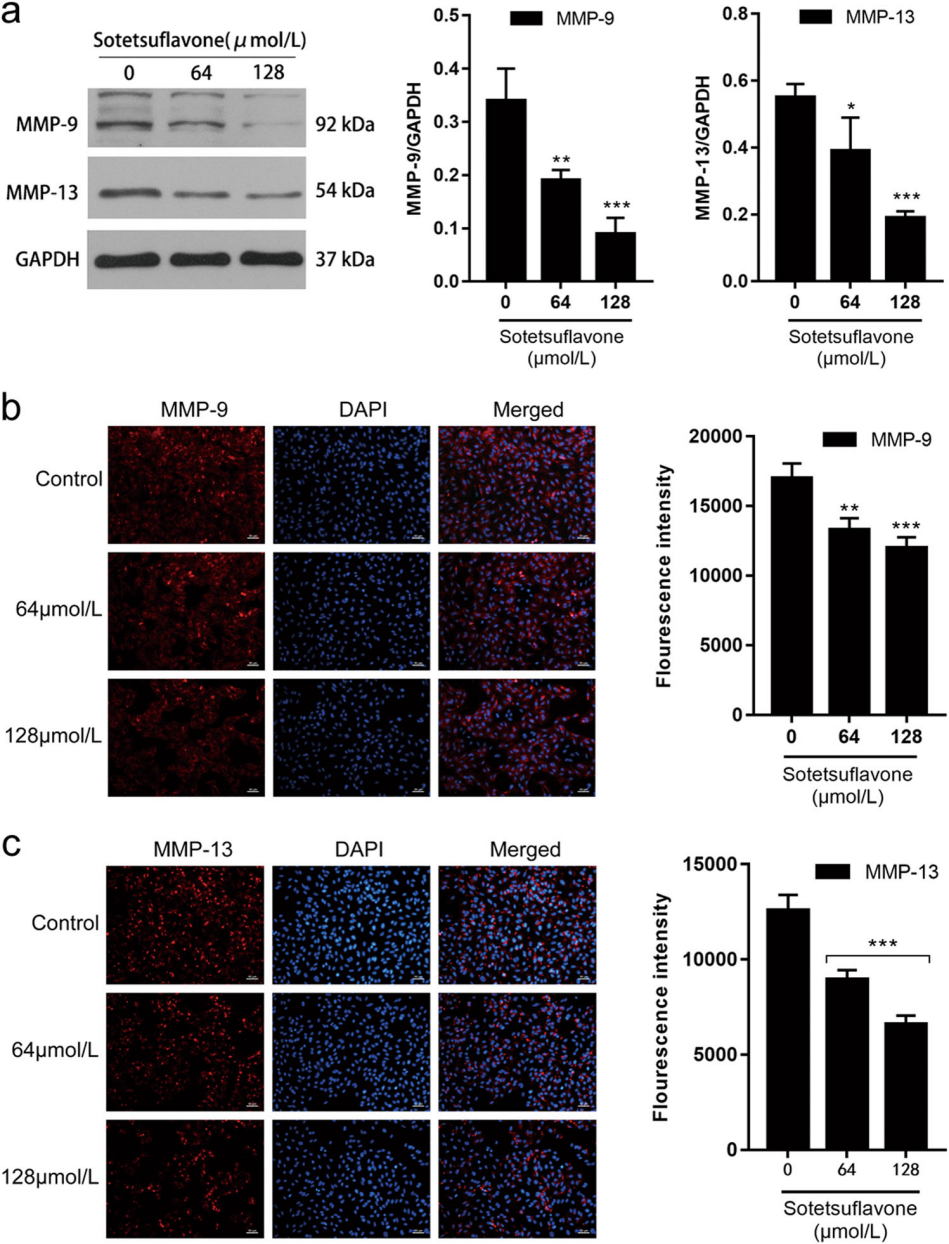
Sotetsuflavone inhibited MMP-9 and MMP-13 expressions. **a** Results from western blotting in detection of the expression of MMP-9 and MMP-13 protein. Using the same sample lysates as used for Fig. 2a, another gel (gel D) was run, the blot was cut horizontally, and probed with anti-MMP-9 and anti-MMP-13. The GAPDH loading control is from gel A, and is the same as shown in Fig. 2a. **b** Immunofluorescence assay detected the expression of MMP-9. Scale bar 50 μm. **c** Immunofluorescence assay used to detect the expression of MMP-13. Scale bar 50 μm. The data in the histogram represented the mean ± SD from three independent experiments (*P < 0.05, **P < 0.01, ***P < 0.001)

HIF-1α can increase the expression of MMPs and thus promote the metastasis of malignant tumors^27^. MMPs play an important role in tumor neovascularization, tumor cell infiltration, and metastatic foci formation^16^. Immunofluorescence and western blot analysis demonstrated that the expression of MMP-9 and MMP-13 was downregulated in A549 cells treated with sotetsuflavone (Fig. 5a). In addition, most of the MMP-9 and MMP-13 detected by the immunofluorescence assay were present in the cytoplasm (Fig. 5b, c).

### Sotetsuflavone inhibited NF-κB pathway activation by downregulating TNF-α

**Fig. 6:**
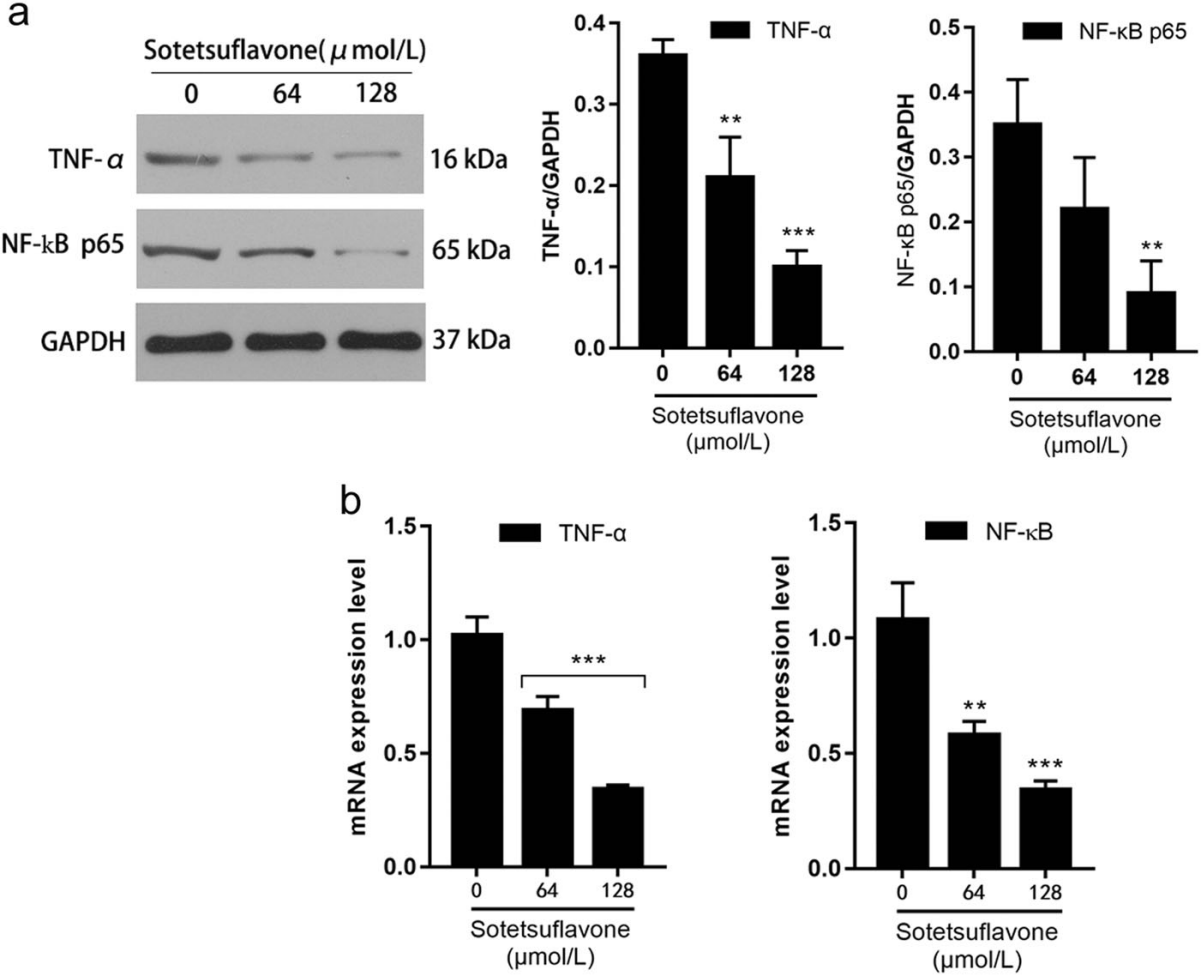
Sotetsuflavone inhibited NF-κB pathway activation by downregulating TNF-α. **a** Results from western blotting on TNF-α and NF-κB protein expression. Using the same sample lysates as used for Fig. 2a, another gel (gel E) was run and probed with anti-TNF-α and anti-NF-κB. The GAPDH loading control is from gel A, and is the same as shown in Fig. 2a. **b** Expression of TNF-α and NF-κB mRNA as estimated by RT-PCR. The data in the histogram represented the mean ± SD from three independent experiments (**P < 0.01, ***P < 0.001)

NF-κB and HIF-1 are related to both gene and protein levels, and it has been suggested that activation of NF-κB and HIF-1α can promote angiogenesis or tumor progression^28^. Our results demonstrated that sotetsuflavone decreased both TNF-α and NF-κB expression in a dose-dependent manner (Fig. 6a, b). Combined with snail expression in Fig. 2a, it was suggested that sotetsuflavone can inhibit the activation of the NF-κB pathway by downregulating TNF-α expression, which inhibited the stability of snail protein by making it easier to degrade and lose its regulation.

### Sotetsuflavone inhibited the PI3K/AKT signaling pathway

**Fig. 7:**
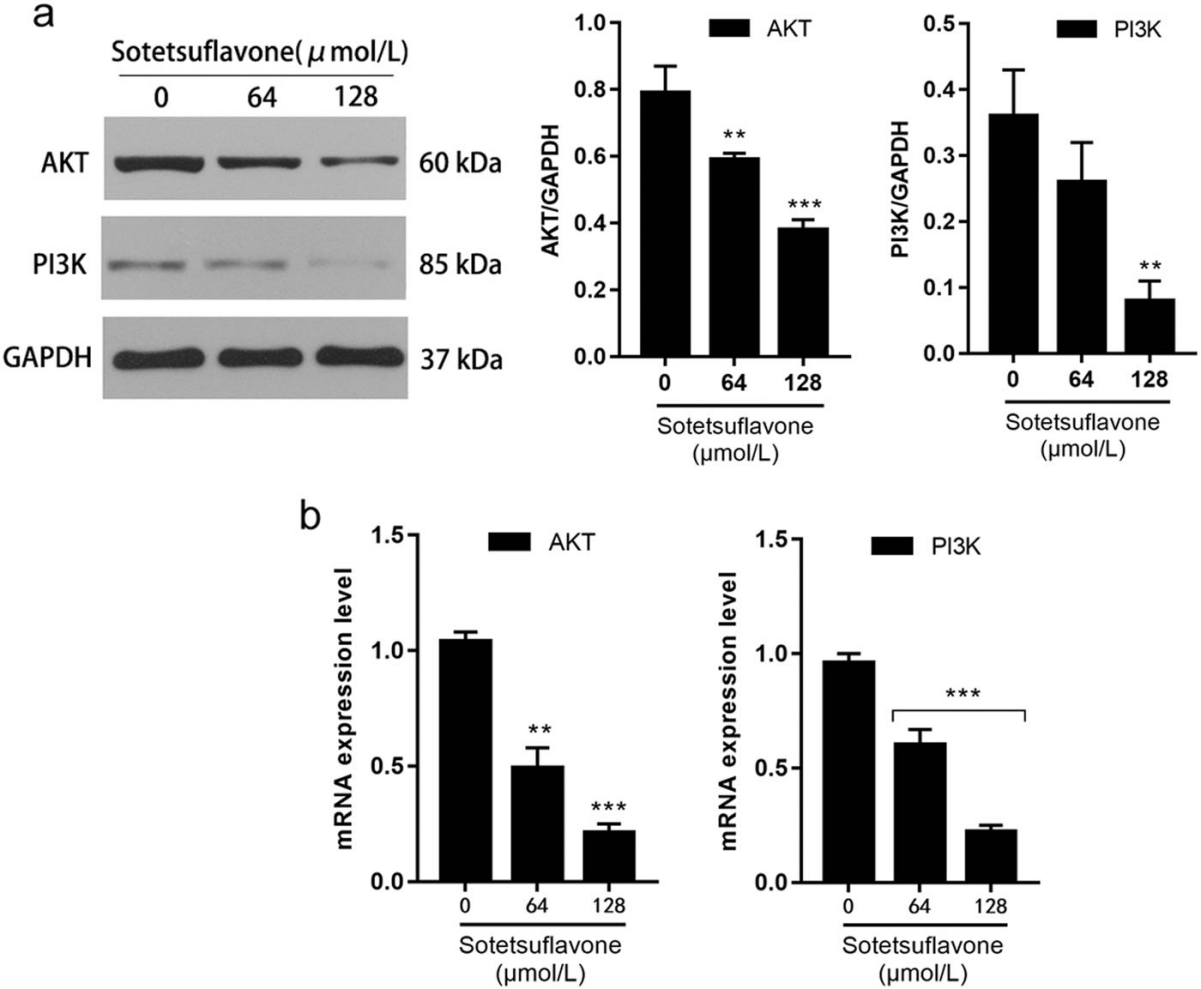
Sotetsuflavone inhibited PI3K/AKT signaling pathway. **a** Results from western blotting showing expression of PI3K and AKT protein. Using the same sample lysates as used for Fig. 2a, another gel (gel F) was run, the blot was cut horizontally, and probed with anti-PI3K and anti-AKT. The GAPDH loading control is from gel A, and is the same as shown in Fig. 2a. **b** Results from RT-PCR shows the expression of PI3K and AKT mRNA. The data in the histogram represented the mean ± SD from three independent experiments (**P < 0.01, ***P < 0.001)

The PI3K/AKT pathway is involved in the regulation of HIF-1α^29^. Western blot analysis and RT-PCR assay demonstrated that the PI3K/AKT pathway was inhibited by sotetsuflavone in a dose- and time-dependent manner along with the inhibition of HIF-1α expression (Figs. 7a, b and 3a, b).

### Diagram of the proposed mechanism by which sotetsuflavone inhibited NSCLC A549 cell invasion and metastasis

**Fig. 8:**
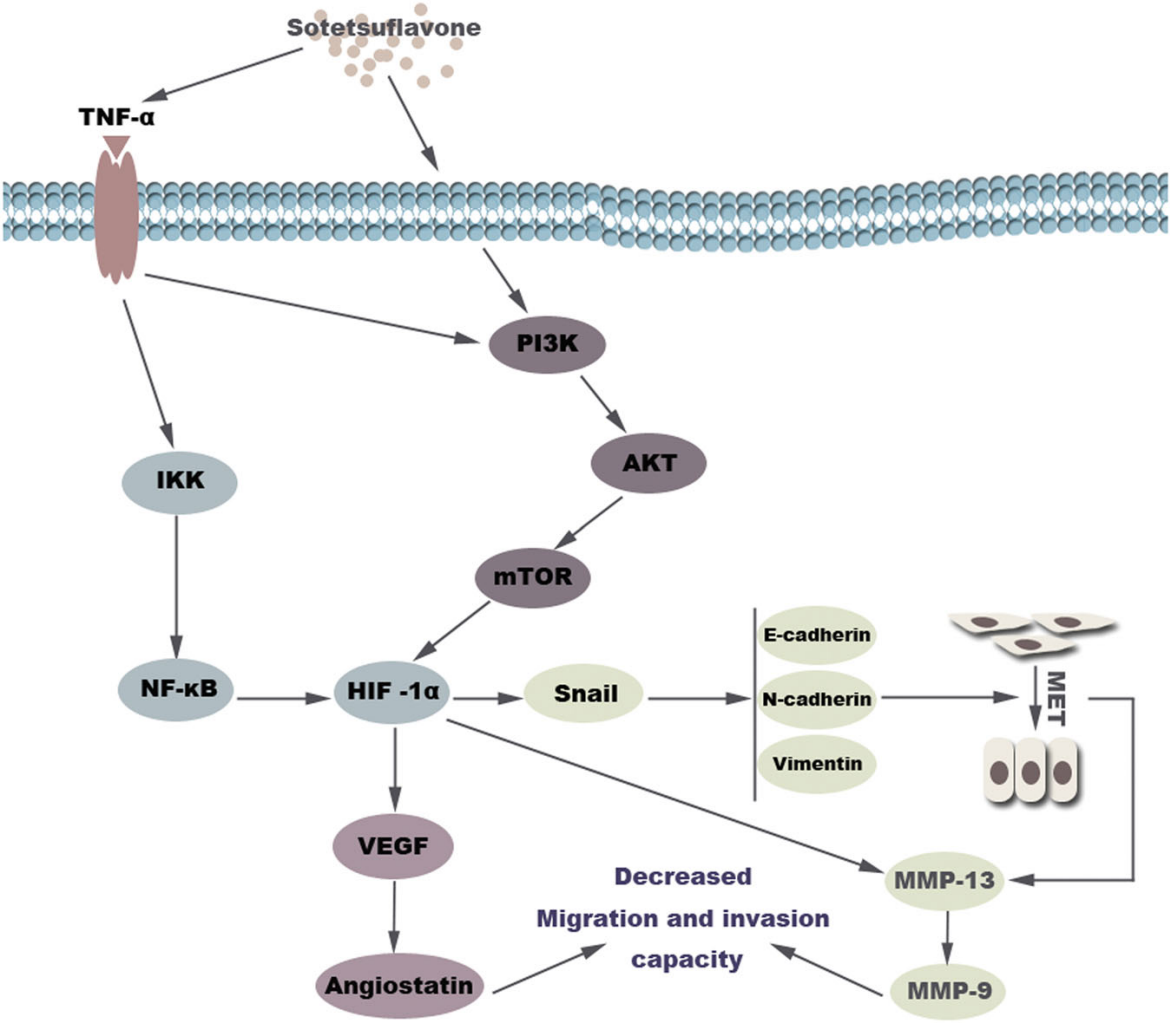
Diagram of the proposed mechanism by which sotetsuflavone inhibits non-small-cell lung cancer A549 cell invasion and metastasis. Sotetsuflavone inhibited the invasion and metastasis of A549 cells by inhibiting EMT and angiogenesis. The anti-transfer effect of sotetsuflavone was mainly through downregulation of HIF-1α by the inhibition of PI3K/AKT and TNF-α/NF-κB signaling pathway in A549 cells, adjusting the whole process

In conclusion, we demonstrated that EMT and angiogenesis play key roles in determining to what extent sotetsuflavone can inhibit the invasion and metastasis of NSCLC A549 cells. More importantly, we discovered that sotetsuflavone could reverse EMT, thereby inhibiting the migration and invasion of A549 cells. This process might inhibit both PI3K/AKT and TNF-α/NF-κB signaling pathways, and downregulate HIF-1α expression. Therefore, HIF-1α may be a potential anticancer target for sotetsuflavone. Figure 8 describes this proposed mechanism.

## Discussion

Sotetsuflavone is a type of double flavonoid extracted from the stem and leaf of *C. revolute*, and has a strong inhibitory activity on DENV-NS5 RdRp^30^. In preliminary work, we have demonstrated that sotetsuflavone can inhibit proliferation and induction of apoptosis in the anti-human lung cancer cell line A549. Based on previous work, we generated the hypothesis that sotetsuflavone could inhibit the migration and invasion of A549 cells by reversing EMT.

In the present study, we discovered that sotetsuflavone can inhibit the migration and invasion of A549 cells (Fig. 1a, b). E-cadherin, N-cadherin, and vimentin are important marker molecules of EMT adjusted by snail^31^. E-cadherin mediates cell adhesion and inhibits tumor invasion and metastasis, and tumor advancement is closely related to its deletion^31^. The expression of N-cadherin in epithelial tissue changes the morphological and biological functions of epithelial cells and transforming epithelial cells into interstitial cells in the EMT process^32^. Vimentin is the major intermediate filament protein found in mesenchymal cells, which regulates cell motility and proliferation, and its expression in epithelial tumors is associated with cell invasiveness^32^. Studies have demonstrated that sulforaphane, quercetin, and other drugs can effectively inhibit EMT via inhibiting the transcription of snail^33^. Therefore, this begs the question: do anti-transfer effects of sotetsuflavone cause this? By detecting EMT-related indicators, we demonstrated that sotetsuflavone inhibited the expression of snail. At the same time, sotetsuflavone significantly increased the expression of the epithelial marker E-cadherin and downregulated the expression of interstitial markers N-cadherin and vimentin (Fig. 2a–c). This induced MET in cells, reducing the ability of tumor cell invasion, and was consistent with the reports of sulforaphane and other drugs. Therefore, these results suggest that sotetsuflavone can inhibit the migration and invasion of A549 cells by regulating EMT.

HIF-1α is an oxygen-dependent transcription factor, and a hypoxic environment promotes the high expression of *HIF-1α* gene in tumor cells and plays an important role in tumorigenesis, development, invasion, metastasis, and apoptosis^13^. VEGF is the most powerful and specific tumor angiogenesis-promoting factor currently known. When VEGF is combined with endothelial cells, it can promote proliferation, migration, and growth of endothelial cells^34^. *VEGF* is an important target gene of HIF-1α. A high expression of HIF-1α upregulates the expression of VEGF, while upregulation of HIF-1α expression can promote the formation of tumor angiogenesis. Additionally, it also plays a role in the growth, invasion, and metastasis of NSCLC cancer tissues, suggesting that blocking HIF-1α/VEGF pathway and inhibiting tumor angiogenesis is an effective anticancer treatment plan^35^. Angiostatin is an isolated angiogenic inhibitor from tumor cells and has a strong inhibitory effects on angiogenesis that inhibit the proliferation and migration of tumor vascular endothelial cells, prevent angiogenesis, promote the primary animal tumor regression, and inhibit the formation of metastases^36^. Therefore, sotetsuflavone should also inhibit tumor angiogenesis. As we predicted, sotetsuflavone can significantly decrease the expression of HIF-1α and VEGF in A549 cells and increase the expression of angiostatin (Figs. 3a, b and 4a–c).

EMT can promote the secretion of MMPs from tumor cells, thereby degrading and destroying the ECM and causing tumor cells to invade and migrate from the primary site^37^. Among them, MMP-9 is a kind of gelatinase in MMPs capable of degrading IV-type collagen in the ECM, which is involved in tumor invasion and metastasis^38,39^. MMP-13 is in the central position of the MMPs’ activation cascade and participates in the degradation of ECM. It also activates MMP-2 and MMP-9 to participate in the development, progression, and invasion of several tumors^40^. We discovered that both MMP-9 and MMP-13 expression were downregulated by sotetsuflavone (Fig. 5a–c).

Multiple signaling pathways are involved in the development and progression of tumors in which the PI3K/AKT pathway is an important factor for tumorigenesis^41^. Both PI3K/AKT and NF-κB pathways are involved in the regulation of HIF-1α^42^. PI3K/AKT is frequently abnormally activated in several human malignancies, and is closely related to tumor recurrence, metastasis, and drug resistance, and has become a hot topic in tumor-targeting drug research^43^. PI3K/AKT signaling pathway regulates the expression of snail and induces the occurrence of EMT^44^. We discovered that the expression of PI3K and AKT were downregulated after sotetsuflavone-treated A549 cells (Fig. 7a, b). In combination with previous results (Fig. 3a, b), it was suggested that the inhibition of HIF-1α expression by sotetsuflavone may be related to the inhibition of PI3K and AKT expression. TNF-α is an important inflammatory factor, and multiple lines of evidence point to the critical role of TNF-α in tumor proliferation, migration, invasion, and angiogenesis^45^. TNF-α has been widely recognized as an important regulator of the tumor microenvironment. The TNF-α/NF-κB pathway plays an important role in tumor cell invasion and metastasis^45^. We examined the effect of sotetsuflavone on the TNF-α/NF-κB pathway. Our results indicated that sotetsuflavone can downregulate TNF-α and inhibit the activation of the NF-κB pathway (Fig. 6a, b). Sotetsuflavone can inhibit epithelial interstitial transformation, invasion, and metastasis of lung cancer cells in A549 cells through both PI3K/AKT and TNF-α/NF-κB pathways.

In summary, sotetsuflavone can inhibit the invasion, migration, and EMT of A549 lung cancer cells. Its mechanism may be involved with the inhibition of PI3K/AKT and TNF-α/NF-κB pathways (Fig. 8). Our results provide a new theoretical basis for the application of sotetsuflavone in molecular targeted therapy of lung adenocarcinoma.

## Materials and methods

### Materials, reagents, and instruments

Sotetsuflavone was isolated in our lab (purity: >98%, high-performance liquid chromatography). CDDP (10 mg/bottle) was purchased from Qilu Pharmaceutical Co., Ltd. (Ji’nan, China). A549 cells (AS6011), phosphate-buffered saline (PBS; AS1025), and 4′,6-diamidino-2-phenylindole (DAPI) dye (AS1075) were purchased from Wuhan Aspen Biotechnology Co., Ltd. (Wuhan, China). Dulbecco’s modified Eagle medium (DMEM) high-glucose medium (SH30022) was purchased from HyClone (Los Angeles, USA). Transwell board bought at Corning Incorporated (Shanghai, China). SDS-polyacrylamide gel electrophoresis (SDS-PAGE) gel preparation kit (AS1012), RIPA total protein lysate (AS1004), BCA protein concentration assay kit (AS1086), and enhanced chemiluminescence detection kit (AS1059) were purchased from Aspen biological (Wuhan, China). StepOne™ Real-Time PCR System was purchased from Life Technologies (Shanghai, China). Anti-angiostatin, GAPDH, VEGF, MMP-9, MMP-13, TNF-α, and snail were purchased from Abcam (Shanghai, China). NF-κB p65, AKT, PI3K, and E-cadherin were purchased at Cell Signaling Technology (Shanghai, China). HIF-1α purchased from Santa Cruz Biotechnology (Shanghai, China). Secondary antibody horseradish peroxidase (HRP)-goat anti-rabbit, HRP-goat anti-mouse, fluorescein isothiocyanate (FITC)-goat anti-rabbit, FITC-goatanti-mouse, CY3-goat anti-rabbit, CY3-goat anti-mouse, and CY3-donkey anti-goat were purchased from Aspen Biological (Wuhan, China); DR-200Bs ELISA was purchased from Wuxi Hiwell Diatek Instruments Co., Ltd. (Wuxi, China). MicroPublisher imaging system (QImaging) was purchased from Shanghai Puch Biotechnology Co. Ltd. (Shanghai, China). CX-21 general optical microscope and IX51 inverted microscope were purchased from Olympus Corporation (Beijing, China).

### Cell culture and drug preparation

A549 cells were cultured in DMEM medium containing 10% fetal bovine serum, 100 U/ml penicillin, and 100 U/ml streptomycin in 5% CO_2_ at 37℃. Logarithmic growth phase cells were used. Sotetsuflavone solution was prepared as a concentrate of 200 mM with dimethylsulfoxide, stored at −20 °C, and diluted to the required concentration with DMEM medium before use. CDDP solution: *N*, *N*-two methyl formamide was first used to prepare a concentrate of 12 mg/ml, and then diluted with DMEM medium to 10 g/ml for the experiment.

### Migration assay

A549 cells in logarithmic growth phase were inoculated into six-well plates at 1 × 10^6^ cells/well. When the cells had adhered to 90%, they were crossed vertically with a 20 μl pipette tip in the middle of each well. Plates were then washed with PBS three times to remove the scraped cells. After adding sotetsuflavone 0 (control group), 32, 64, and 128 μmol/L, the culture was allowed to grow in 5% CO_2_ at 37 ℃. CDDP was used as a positive control drug. The scratch width changes of sotetsuflavone and CDDP on A549 cells for 24 h were observed under a microscope, and scratch-healing rate (%) = (initial scratch width − scratch width of specified time)/initial scratch width × 100%.

### Invasion assay

We took 50 μl of Matrigel and the 1:3 of culture medium dilution liquid and added it to the Transwell chamber. We then collected 200 μl 105 cells/ml cell suspension (serum-free medium) in a Transwell chamber, and added 500 μl/well of 0, 32, 64, 128 μmol/l of sotetsuflavone and 10 μg/ml of cisplatin, and incubated at 37 °C for 24 h. The medium was removed and the A549 cells in the upper chamber were wiped with cotton swabs. Cells were fixed in formaldehyde for 20 min, dried, and stained with the solution of PBS 0.01% crystal violet for 10 min. Crystal violet was washed from the surface, and five images were taken at random under an inverted microscope, and the number of transmembrane cells in the photographs were counted.

### Reverse transcription-PCR assay

A549 cells were treated with different concentrations (0, 64, and 128 μmol/l) for 24 h, then total RNAs was extracted using the Trizol cell lysis method. After measuring the purity and concentration, then added reverse transcription reagent, and transcribed at 37 °C for 15 min. The cDNA was reacted with the upstream and downstream primers and SYBR Green fluorescent dye was added for real-time quantitative PCR. The following PCR program was used: denaturation 95 °C for 1 min, followed by 40 cycles consisting of denaturation at 95 °C for 15 s, annealing at 58 °C for 20 s, and extension 72 °C for 45 s, A melting curve analysis was applied to assess the specificity of the amplified PCR products. The PCR primers were as follows: VEGF, forward 5′-GAACTTTCTGCTGTCTTGGGTG-3′ and reverse 5′-GGCAGTAGCTGCGCTGATAG-3′; TNF-α, forward 5′-CTCTTCTCCTTCCTGATCGTGG-3′ and reverse 5′-CTTGTCACTCGGGGTTCGAG-3′; NF-κB, forward 5′-CGCATCCAGACCAACAACA-3′ and reverse 5′-TGCCAGAGTTTCGGTTCAC-3′; vimentin, forward 5′-CAGGCAAAGCAGGAGTCCAC-3′ and reverse 5′-TATTCACGAAGGTGACGAGCC-3′; snail, forward 5′-GGCTGCTACAAGGCCATGTC-3′ and reverse 5′-ACTCTTGGTGCTTGTGGAGCA-3′; N-cadherin, forward 5′-AGAGGCAGAGACTTGCGAAAC-3′ and reverse 5′-ACACTGGCAAACCTTCACGC-3′; PI3K, forward 5′-GTCCTATTGTCGTGCATGTGG-3′ and reverse 5′-TGGGTTCTCCCAATTCAACC-3′; AKT, forward 5′- TTCTATGGCGCTGAGATTGTGT-3′ and reverse 5′-GCCGTAGTCATTGTCCTCCAG-3′; and GAPDH, forward 5′-GGTCGGAGTCAACGGATTTG-3′ and reverse 5′-GGAAGATGGTGATGGGATTTC-3′. The amount of each target gene was quantified by the comparative CT method using GAPDH as the normalization control.

### Western blot analysis

A549 cells were treated with 0, 64, 128 μmol/l sotetsuflavone for 24 h, and total protein was extracted with RIPA lysis buffer. The protein samples were separated by SDS-PAGE and then transferred to a polyvinylidene fluoride membrane, and closed 1 h with TBST solution containing 5% skim milk powder, we added the primary antibodies overnight at 4℃. After three washes of 10 min each in TBST, the membranes were incubated with HRP-conjugated secondary antibodies for 1.5 h and subsequently washed again. An ECL detection system was used to visualize immunoreactive bands. Equal protein sample loading was monitored by probing the same membrane with antibodies against GAPDH.

### Immunofluorescent staining

A549 cells were cultivated in 24-well plates and treated by sotetsuflavone. Then the cells were washed with PBS three times for 5 min and fixed with 4% paraformaldehyde for 30 min. After permeabilization with 0.1% Triton X-100, cells were blocked with 5% bovine serum albumin for 1 h, and incubated with primary antibodies at 4 °C overnight. After washing with PBS, the cells were incubated with secondary antibodies corresponding to the primary antibody for 50 min at 37 °C, and counterstained with DAPI. Images were taken using a MicroPublisher imaging system.

### Statistical analysis

The experimental data were processed by SPSS 20.0 statistical software. All experiments were repeated at least three times. The measurement data were expressed as mean ± standard deviation (*x* ± *s*). The one-way analysis of variance was used to analyze the variance, when the variance was homogeneous, with LSD, SNK test, conversely, use Dunnett T3 test. *P*-values <0.05 were considered statistically significant.
